# Health Literacy and Access to Care among Patients with and Without Traumatic Brain Injury: An All of Us Analysis

**DOI:** 10.1177/10538135251393519

**Published:** 2025-11-11

**Authors:** Richard Cook, Kathleen R Ran, Vikas N Vattipally, Gorbachev Jowah, Naren Gundapaneni, Rajiv Dharnipragada, Jose Suarez, John Williams, Tej Azad

**Affiliations:** 1 Department of Neurosurgery, McGovern Medical School at UTHealth Houston, Houston, TX, USA; 2 Department of Neurosurgery, Johns Hopkins Hospital, Baltimore, MD, USA

**Keywords:** “traumatic brain injury”, “health literacy”, “healthcare access”, “socioeconomic status”, “All of us”

## Abstract

**Background:**

Health literacy, which enables patients to effectively navigate the healthcare system, is associated with improved patient outcomes. Patients with traumatic brain injury (TBI) may be uniquely susceptible to gaps in health literacy and access to care, particularly for those of vulnerable socioeconomic status.

**Methods:**

Using the National Institutes of Health's (NIH) All of Us Research Program, we analyzed survey responses among matched participants with and without TBI (*N* = 2,330 and *N* = 11,562, respectively) via nearest-neighbor propensity score matching. Chi-squared tests compared responses between racial/ethnic and income groups among those with TBI. Multivariable binary and logistic regression compared responses between matched participants with and without TBI.

**Results:**

Respondents with TBI were more likely to report issues related to health literacy and financial barriers to care. Multivariable analyses revealed participants with TBI reported increased difficulty understanding medical forms (OR 1.31, 95% CI 1.18-1.45, *P* *<* 0.001) and difficulty affording emergency care (OR 1.55, 95% CI 1.26–1.90, *P* *<* 0.001) when compared to those without TBI.

**Conclusion:**

Respondents with history of TBI, per electronic health records, are more likely to report issues pertaining to health literacy and access to care. Among those with TBI, historically underserved populations are also more likely to report issues relating to health literacy and financial barriers to care, though TBI did not consistently modify the effects of race/ethnicity or income. Multidisciplinary efforts to address these limitations are necessary.

## Introduction

Health literacy, which is broadly defined as the ability to meet the demands of the healthcare system, allows patients to effectively navigate their care and receive appropriate treatment (Baker, 2006; Levy & Janke, 2016; Rasu et al., 2015; Sørensen et al., 2012). Yet over one-third of adults in the United States (36%) demonstrate below-basic health literacy (Magnani et al., 2018). Examples of health literacy include the ability to follow medical instructions and schedule appointments (Mårtensson & Hensing, 2012). Patients with higher health literacy are more likely to adhere to treatment (Kalichman et al., 2008; Kripalani et al., 2006), while low health literacy is associated with poorer outcomes, poorer resource utilization, and worse physical and mental health (Arsenović et al., 2023; Baker et al., 2007; Berkman et al., 2011; Francis et al., 2007).

Low health literacy is associated with various socioeconomic factors. Lower educational attainment, lower household income, unemployment, older age, male gender, and racial/ethnic minority status have all been associated with lower health literacy (Chen et al., 2024; Gao et al., 2025; García-García & Pérez-Rivas, 2022; Svendsen et al., 2020; Yang et al., 2021). Additionally, financial barriers, which have grown with rising healthcare costs, may further compound the challenges of navigating health information and accessing appropriate care (Farmanova et al., 2018; Megan, 2023; Stormacq et al., 2023).

These concerns are especially pertinent for those with traumatic brain injury (TBI), for which an estimated 1.1 million Americans are treated each year (Corrigan et al., 2010). The effects of TBI, such as functional, cognitive, behavioral, and social impairments, may last as long as 30 years and require ongoing medical care throughout a patient's life (Brett et al., 2023; Dams-O’Connor et al., 2023). Studies have demonstrated that socioeconomically disadvantaged populations are at higher risk of TBI (Bruns & Hauser, 2003; Haines et al., 2019; Kraus et al., 1986). Among patients with TBI, lower health literacy is associated with worse perceived physical health and more depressive symptoms (Pappadis et al., 2023). Nearly one-third of patients with TBI demonstrate marginal or inadequate health literacy, though how TBI contributes to this is unclear (Hicks et al., 2024; Sander et al., 2024). While systematic reviews on TBI and health literacy are limited, TBI has been associated with decreased reading ability and discourse deficits (Hill et al., 2018; Pei & O’Brien, 2021). Additional healthcare costs and functional deficits from TBI may exacerbate existing barriers to care and contribute to a lower health-related quality of life (Andelic et al., 2016; Cuthbert et al., 2015; Eliacin et al., 2018; Gorgoraptis et al., 2019; Humphreys et al., 2013; Rubin, 2020). The consequences are often particularly severe among racial and ethnic minorities as well as low-income patients (Haines et al., 2019; Kelly et al., 2022; Liao et al., 2012; Salik et al., 2022). Despite their importance, health literacy and access to care among patients with TBI are underexplored. Understanding the relationship between TBI, health literacy, and access to care is critical to identifying and addressing barriers to care.

This analysis explores the associations between TBI and individual health literacy and access to care using survey responses from the National Institutes of Health's (NIH) All of Us Research program. We further investigate how TBI relates to health literacy and access to care across income and racial/ethnic groups. Elucidating health literacy among patients with TBI may provide a framework for rectifying gaps in care, especially for socioeconomically disadvantaged patients.

## Materials and Methods

### Dataset

Data were retrospectively analyzed from the National Institute of Health's (NIH) All of Us Research Program, which is a patient-enrolled program that collects electronic health record (EHR) data and responses to various surveys. Namely, this analysis utilized “The Basics,” “Overall Health,” and “Health Care Access and Utilization” surveys. These surveys were selected on the basis that they assess respondents’ ability to navigate health materials and potential financial barriers to healthcare. These surveys provided standardized items across participants and enabled coding into SNOMED concepts, ensuring comparability between groups. Data collected by All of Us are accessible via their Research Workbench, which requires an institutional Data Use and Registration Agreement (DURA) and a verified Login.gov ID (*All of Us Research Program Protocol*, 2020).

### Cohort Definition

This study utilized two cohorts: one consisting of participants with a history of TBI, per the participants’ EHR data, and one of participants without a history of TBI. Demographic information, including race, ethnicity, sex, age, education level, income, marital status, employment status, and homeowner status, was collected for each participant. Only respondents who filled out both the “Overall Health” and “Health Care Access and Utilization” surveys were included. All participants in All of Us are self-enrolled. Enrollment started in May 2017, and the respondent cohort used for analysis was created in March 2024.

Participants with TBI were defined as having “traumatic brain injury” listed as a condition per All of Us’ standard vocabulary code (SNOMED code 127295002), which is converted from International Classification of Diseases 9^th^ and 10^th^ edition codes. These participants were included in the TBI cohort. Participants without “traumatic brain injury” listed as a condition were included in the non-TBI cohort.

### Variable Definitions

Questions from the “Overall Health” survey, which is publicly available under “Surveys” within All of Us’ data browser, were used to assess respondents’ ability to navigate health material. For respondents with multiple entries, the most recent survey responses were used for analysis. The “Overall Health” survey assessed how often participants needed assistance with medical forms, how often participants experienced difficulty understanding written information about their medical condition, and participants’ confidence levels in independently completing medical forms (question IDs 1585772, 1585778, 1585766, respectively). For each of these three questions, participants can select five varying degrees of frequency or severity (e.g., “never,” “rarely,” “sometimes,” “often,” or “always”), or, as with any question in the survey, skip the question.

Responses to the “Health Care Access and Utilization” survey were used to assess access to medical care. This survey queried about participants’ ability to afford medical care, including emergency care (question ID 43528663), follow-up care (question ID 43530409), a healthcare provider (question ID 43529901), dental care (question ID 43528662), and specialist care (question ID 43530412). Whether participants delayed care due to insurance copayment (question ID 43530583), a deductible (question ID 43530585), out-of-pocket payment (question ID 43530584), childcare (question ID 43529903), or inability to take time off work (question ID 43529905) was also assessed. Additionally, responses to whether participants delayed (question ID 43530415), skipped (question ID 43530416), or took less than the prescribed amount of a prescription (question ID 43530417) were analyzed. All of these were yes/no questions regarding whether they had experienced these barriers to care in the last 12 months. Finally, levels of concern about paying for medical care at the time of filling out the survey were assessed, for which participants had three options to rank their level of concern (question ID 43530557).

To account for potential confounders in the relationship between TBI and health literacy, sociodemographic and economic variables identified in prior studies as determinants of health literacy were included in analysis. Accordingly, demographic information from the “The Basics” survey was collected to ensure comparability between the two cohorts. Employment status (question ID 1585952), annual income (question ID 1585375), educational attainment (question ID 1585940), marital status (question ID 1585892), and homeowner status (question ID 1585370) were assessed from this survey. Sex and race/ethnicity were determined per EHR data. Race and ethnicity were combined to create a race/ethnicity variable with three categories: Hispanic, non-Hispanic Black, or non-Hispanic White. Participants’ education level was classified into four categories: less than high school, high school (or equivalent), some college, and college degree or higher. Employment status was dichotomized as either employed for wages or unemployed/other. Informative nonresponses (e.g., “skip,” “don’t know,” “prefer not to answer”) were collapsed into a single “skip” category, while all other responses were reported as provided. Notably, though annual income was reported as provided, it was divided into four approximate quartiles for the purposes of chi-squared analysis: less than $35,000, $35,000 to $74,999, $75,000 to $99,999, and $100,000 or more per year.

### Statistical Analysis

Analysis was conducted in accordance with the All of Us’ Ethical Conduct of Research Policy and Institutional Review Board (IRB). Matching among the TBI and non-TBI cohorts based on sex, age, race/ethnicity, employment, education, income, marital status, and homeowner status was performed using the MatchIt package in R (Ho et al., 2023). Nearest-neighbor matching without replacement was performed at a 1:5 ratio (one participant with TBI matched to up to five participants without TBI), with a caliper of 0.2 standard deviations of the logit of the propensity score to restrict poor matches. Exact matching was required on sex and race/ethnicity to ensure balance on these key demographic factors. Bivariable analysis was performed using a chi-squared test to compare survey responses among racial/ethnic groups and income groups among respondents with TBI to assess how health literacy and access to care vary by race/ethnicity and income group. Multivariable analysis, using respondents’ age, sex, race/ethnicity, employment, income, education, marital status, and homeowner status as covariates, was performed using ordinal logistic regression to assess the association of TBI with ordinal survey responses among matched participants with and without TBI. This method was chosen because such survey questions are structured with ordered categorical scales (e.g., frequency or level of agreement), and the proportional odds framework preserves the ordinal information without requiring artificial dichotomization (Wieditz et al., 2024). Binary logistic regression was used to determine the association of TBI with dichotomous (yes/no) survey responses. One question (question ID 43530557, assessing the degree of being worried about paying for healthcare) was collapsed to a binary variable for the purpose of binary logistic regression since it had only three levels of responses. For this question, binary regression was used to assess the association between being “very” worried or not “very” worried. Informative nonresponses were excluded from regression analysis, and sensitivity analysis was performed as described below in *Missing data*. Finally, to assess effect modification, interaction terms between TBI and race/ethnicity, and between TBI and income, were included in regression models for each survey question. All data analysis was conducted in All of Us’ Researcher Workbench, an online platform for accessing All of Us data.

### Missing Data

Missing survey data were categorized as either noninformative nonresponses (e.g., variables recorded as missing [NA]) or informative nonresponses (e.g., participant-selected responses of “skip” or “don’t know”). Respondents with noninformative nonresponses were excluded prior to matching to ensure valid comparisons between respondents with and without TBI. After matching, informative nonresponses were excluded from multivariable regression analysis but were included and reported separately for chi-squared analysis. A summary of missingness patterns is provided in Supplemental Table 1.

To assess robustness, best- and worst-case sensitivity analyses were performed to confirm the significance of positive findings. In these analyses, informative nonresponses were reclassified as the least severe response option (best-case) or the most severe response option (worst-case), and the models were re-estimated accordingly.

### Correction for Multiple Hypotheses

For this analysis, 15 prespecified survey outcomes were evaluated. To control for multiple testing, Bonferroni correction for 15 comparisons was applied, setting the significance threshold at *P* < 0.0033.

## Results

### Cohort Overview

A total of 13,892 participants were included in our analysis. Demographic characteristics of matched participants without TBI (*N* = 11,562) and with TBI (*N* = 2,330) are summarized in Table 1. The average age for participants with and without TBI was 54.3 (SD = 16.4) and 54.6 (SD = 16.5) years, respectively. The majority of participants were female, comprising 67.0% of each group. Further, the majority, 82.6%, of all respondents were non-Hispanic White. The most commonly reported income was $50,000–74,999 per year, which comprised 15.5% of all participants. Overall, most participants had a college education or more (56.3%).

**Table 1.** Demographic Characteristics of all Participants, as Well as Those with and Without TBI.

Factor	All Participants	Participants without TBI	Participants with TBI	*P*-Value
*N*	13,892	11,562	2,330	—
Age (years) (mean ± SD)	54.6 ± 16.5	54.6 ± 16.5	54.3 ± 16.4	0.425
Sex (*N*) (%)				
*Female*	9,314 (67.0%)	7,752 (67.0%)	1,562 (67.0%)	>0.999
*Male*	4,490 (32.3%)	3,738 (32.3%)	752 (32.3%)	0.978
*Other*	88 (0.6%)	72 (0.6%)	16 (0.7%)	0.832
Race/ethnicity (*N*) (%)				
*Black, non-Hispanic*	1,206 (8.7%)	1,005 (8.7%)	201 (8.6%)	0.950
*White, non-Hispanic*	11,474 (82.6%)	9,561 (82.7%)	1,913 (82.1%)	0.512
*Hispanic*	1,212 (8.7%)	996 (8.6%)	216 (9.3%)	0.325
Employment (*N*) (%)				
*Employed*	6,139 (44.2%)	5,111 (44.2%)	1,028 (44.1%)	0.958
*Unemployed/other*	6,748 (48.6%)	5,613 (48.5%)	1,135 (48.7%)	0.902
*Skip*	1,005 (7.2%)	838 (7.2%)	167 (7.2%)	0.926
Income (*N*) (%)				
*<$10,000/yr*	952 (6.9%)	786 (6.8%)	166 (7.1%)	0.600
*$10,000–$24,999/yr*	1,791 (12.9%)	1,498 (13%)	293 (12.6%)	0.641
*$25,000–$34,999/yr*	1,120 (8.1%)	927 (8%)	193 (8.3%)	0.698
*$35,000–$49,999/yr*	1,356 (9.8%)	1,128 (9.8%)	228 (9.8%)	0.996
*$50,000–$74,999/yr*	2,152 (15.5%)	1,797 (15.5%)	355 (15.2%)	0.733
*$75,000–$99,999/yr*	1,494 (10.8%)	1,233 (10.7%)	261 (11.2%)	0.467
*$100,000–$149,999/yr*	1,835 (13.2%)	1,540 (13.3%)	295 (12.7%)	0.411
*$150,000–$199,999/yr*	839 (6%)	695 (6%)	144 (6.2%)	0.791
*>$200,000/yr*	853 (6.1%)	710 (6.1%)	143 (6.1%)	>0.999
*Skip*	1,500 (10.8%)	1,248 (10.8%)	252 (10.8%)	>0.999
Education (*N*) (%)				
*Less than high school*	320 (2.3%)	257 (2.2%)	63 (2.7%)	0.181
*High school*	1,706 (12.3%)	1,423 (12.3%)	283 (12.1%)	0.855
*Some college*	3,912 (28.2%)	3,249 (28.1%)	663 (28.5%)	0.748
*College or more*	7,818 (56.3%)	6,517 (56.4%)	1,301 (55.8%)	0.655
*Skip*	136 (1%)	116 (1%)	20 (0.9%)	0.594
Marital status (*N*) (%)				
*Married*	6,754 (48.6%)	5,632 (48.7%)	1,122 (48.2%)	0.640
*Divorced*	2,181 (15.7%)	1,810 (15.7%)	371 (15.9%)	0.769
*Never married*	2,778 (20%)	2,312 (20%)	466 (20%)	>0.999
*Separated*	343 (2.5%)	280 (2.4%)	63 (2.7%)	0.467
*Living with partner*	851 (6.1%)	707 (6.1%)	144 (6.2%)	0.942
*Widowed*	771 (5.5%)	643 (5.6%)	128 (5.5%)	0.936
*Skip*	214 (1.5%)	178 (1.5%)	36 (1.5%)	>0.999
Homeowner status (*N*) (%)				
Owner	8,708 (62.7%)	7,249 (62.7%)	1,459 (62.6%)	0.962
Renter	4,860 (35%)	4044 (35%)	816 (35%)	0.986
Other/skip	324 (2.3%)	269 (2.3%)	55 (2.4%)	0.981

### Association Between TBI and Self-Reported Health Literacy and Healthcare Access

Multivariate ordinal logistic regression demonstrated a significant association between TBI and reporting issues related to difficulty navigating health material, as shown in Table 2. TBI was significantly associated with difficulty understanding health material (OR 1.31, 95% CI 1.18–1.45, *P* < 0.001) and requiring assistance with health forms (OR 1.24, 95% CI 1.12–1.36, *P* *<* 0.001). Associations with difficulty understanding and requiring assistance were consistent throughout sensitivity analysis, as shown in Supplemental Table 2.

**Table 2.** Association of TBI with Difficulty Navigating Health Material and Access to Care as Determined by Multivariable Ordinal and Binary Logistic Regression among Matched Participants with and Without TBI.

Variable	OR	95% Confidence Interval		*P*-Value
Difficulty understanding	1.31	1.18	1.45	*<0.001*
Require assistance	1.24	1.12	1.36	*<0.001*
Confidence	0.89	0.81	0.99	0.030
Can’t afford…				
*emergency care*	1.55	1.26	1.90	*<0.001*
*follow-up care*	1.16	0.98	1.37	0.080
*dental care*	1.03	0.91	1.16	0.630
*a healthcare provider*	0.99	0.82	1.19	0.910
*a specialist*	1.12	0.96	1.3	0.150
Delayed care due to…				
*copay*	1.06	0.91	1.24	0.460
*childcare*	1.28	0.99	1.65	0.060
*deductible*	1.01	0.87	1.18	0.900
*paying out of pocket*	0.94	0.83	1.06	0.340
*time off work*	1.1	0.94	1.28	0.240
*transportation*	1.48	1.27	1.73	*<0.001*
Took less medications	1.16	0.99	1.34	0.060
Delayed medications	1.22	1.07	1.39	*0*.*002*
Skipped medications	1.26	1.08	1.48	0.004
Worried about paying	1.05	0.91	1.21	0.520

Associations between TBI and healthcare access are also reported in Table 2. TBI was associated with inability to afford emergency care (OR 1.55, 95% CI 1.26–1.90, *P* *<* 0.001), delayed care due to transportation (OR 1.48, 95% CI 1.27–1.73, *P* *<* 0.001). Additionally, TBI was associated with delaying filling medications due to cost (OR 1.22, 95% CI 1.08–1.39, *P* *=* 0.002). These associations were consistent throughout best- and worst-case sensitivity analysis, as shown in Supplemental Table 2.

### Impact of Race and Ethnicity on Self-Reported Health Literacy and Healthcare Access among Participants with TBI

Table 3 compares health literacy among participants with TBI based on race/ethnicity. Non-Hispanic White participants were more likely to report never experiencing difficulty understanding health material (*P* < 0.001), never requiring assistance with medical forms (*P* < 0.001), and being extremely confident with medical forms (*P* < 0.001). In contrast, both Hispanic and non-Hispanic Black participants reported higher rates of always experiencing difficulty understanding health material (*P* < 0.001) and always requiring assistance with medical forms (*P* < 0.001). These results were consistent throughout best- and worst-case sensitivity analysis, shown in Supplemental Table 3.

**Table 3.** Difficulty Navigating Health Material and Access to various Aspects of Healthcare by Race and Ethnicity among Participants with TBI.

Variable	Black, non-Hispanic (174)	Hispanic (175)	White, non-Hispanic (1,734)	*P*-Value
Difficulty Understanding (%)				
*Never*	61.5	65.7	74.7	*<0.001*
*Occasionally*	20.1	12.0	17.4	0.110
*Sometimes*	10.9	9.7	5.1	*0*.*001*
*Often*	4.0	5.7	1.3	*<0.001*
*Always*	3.4	5.1	0.5	*<0.001*
*Skip*	0.0	1.7	1.0	0.261
Assistance with Medical Forms (%)				
*Never*	60.9	56.0	70.4	*<0.001*
*Occasionally*	17.8	19.4	18.5	0.925
*Sometimes*	12.6	11.4	6.1	*<0.001*
*Often*	4.0	4.0	3.2	0.726
*Always*	2.9	8.6	1.0	*<0.001*
*Skip*	1.7	0.6	0.9	0.364
Confidence with Medical Forms (%)				
*Not at all*	1.1	1.7	0.5	0.085
*A little bit*	2.3	2.3	0.8	0.028
*Somewhat*	9.8	8.0	4.7	0.006
*Quite a bit*	19.5	28.6	18.6	0.006
*Extremely*	66.7	59.4	74.7	*<0.001*
*Skip*	0.6	0.0	0.6	0.717
Can’t afford… (%)				
*emergency care*	9.2	10.3	5.1	0.004
*follow-up*	12.6	16.6	7.6	*<0.001*
*a healthcare provider*	13.2	10.9	5.3	*<0.001*
*dental care*	23.6	25.1	17.9	0.018
*specialist care*	14.4	18.9	9.6	*<0.001*
Delayed care due to… (%)				
*copay*	12.1	15.4	9.3	0.023
*childcare*	5.7	8.6	2.8	*<0.001*
*deductible*	10.9	15.4	10.0	0.085
*paying out of pocket*	14.9	18.3	17.1	0.690
*time off work*	11.5	18.3	10.3	0.006
*transportation*	22.4	16.0	9.5	*<0.001*
Worried about paying (%)				
*Somewhat*	23.6	35.4	37.7	*0*.*001*
*Very*	14.4	26.3	11.6	*<0.001*
Took less medication	12.6	15.4	10.4	0.097
Skipped medication	12.1	14.3	9.5	0.093
Delayed medication	17.8	17.7	15.1	0.442

Table 3 also compares access to healthcare among participants with TBI based on race/ethnicity. Non-Hispanic Black and Hispanic participants more frequently reported not being able to afford follow-up care (*P* *<* 0.001), a healthcare provider (*P* *<* 0.001), and specialist care (*P* *<* 0.001). Non-Hispanic Black and Hispanic participants also more frequently reported delaying care due to childcare (*P* *<* 0.001) and lack of transportation (*P* *<* 0.001). Non-Hispanic White participants were more likely to report being somewhat worried about paying for medical care (*P* *=* 0.001), while Hispanic patients were most likely to report being very worried (*P* *<* 0.001). These results were consistent throughout sensitivity analysis, shown in Supplemental Table 3. Additionally, Supplemental Table 4 shows a similar trend among respondents without TBI, in which fewer non-Hispanic White respondents reported issues related to health literacy and access to care, and more Hispanic and non-Hispanic Black respondents reported these issues.

Table 4 shows how difficulty navigating health materials varies by income group among respondents with TBI. Participants in the highest income bracket, $100,000 per year or more, were most likely to report never experiencing difficulty understanding health material (*P* < 0.001), never needing assistance with medical forms (*P* < 0.001), and being extremely confident with medical forms (*P* < 0.001). Further, the lowest income bracket, less than $35,000 per year, was more likely to report always experiencing difficulty understanding health material (*P* < 0.001) and always needing assistance with medical forms (*P* < 0.001). Notably, when informative nonresponses were reclassified as the most severe response in sensitivity analysis, the p-value comparing responses for always experiencing difficulty understanding health material became non-significant (*P* = 0.004), as shown in Supplemental Table 5. All other mentioned findings were consistent throughout sensitivity analysis. Additionally, Supplemental Table 6 shows a similar trend among those without TBI, in which respondents earning $100,000 per year or more were less likely to report issues related to health literacy, and those earning less than $35,000 per year were more likely to report these issues.

**Table 4.** Difficulty Navigating Health Material by Income among Participants with TBI.

Variable	*<*$35,000/yr (657)	$35,000–74,999/yr (583)	$75,000–99,999/yr (261)	≥$100,000/yr (582)	*P*-value
Difficulty Understanding (%)					
*Never*	58.0	74.6	77.8	85.6	*<0.001*
*Occasionally*	21.8	18.2	16.9	11.2	*<0.001*
*Sometimes*	12.5	4.1	2.7	2.1	*<0.001*
*Often*	4.1	1.4	1.1	0.3	*<0.001*
*Always*	2.6	0.7	0.4	0.2	*<0.001*
*Skip*	1.1	1.0	1.1	0.7	0.878
Assistance with Medical Forms (%)					
*Never*	53.9	71.2	76.6	78.4	*<0.001*
*Occasionally*	22.8	18.2	16.5	14.8	*0.003*
*Sometimes*	13.5	5.1	3.1	3.4	*<0.001*
*Often*	4.7	3.4	1.9	2.2	0.051
*Always*	4.0	1.0	1.1	0.5	*<0.001*
*Skip*	1.1	1.0	0.8	0.7	0.941
Confidence with Medical Forms (%)					
*Not at all*	1.2	0.9	0.0	0.2	0.063
*A little bit*	2.4	0.5	0.8	0.2	*0.001*
*Somewhat*	11.3	5.0	1.5	1.0	*<0.001*
*Quite a bit*	24.4	19.9	21.1	12.9	*<0.001*
*Extremely*	60.0	73.1	75.9	85.6	*<0.001*
*Skip*	0.8	0.7	0.8	0.2	0.422

### Association Between Interaction of TBI with Race/Ethnicity and Income with Self-Reported Health Literacy and Healthcare Access

Supplemental Tables 7 and 8 show how the interaction of TBI with race/ethnicity and income, respectively, is associated with difficulty navigating health materials and financial barriers to care, as determined via multivariable ordinal and binary logistic regression. Only delaying care due to transportation was associated with TBI and earning more than $100,000 per year (OR 2.12, 95% CI 1.29–3.50, *P* = 0.003). There were no other statistically significant effect modifications.

## Discussion

Comparison between matched participants with and without EHR history of TBI demonstrated those with history of TBI are significantly more likely to report survey responses associated with low health literacy and more likely to report various financial barriers to care. Further, Hispanic and non-Hispanic Black respondents and respondents in the lower income brackets were more likely to report these issues when compared to non-Hispanic White and higher income respondents, respectively. However, history of TBI did not significantly modify the effect of race/ethnicity or income regarding reporting issues related to health literacy or financial barriers to care, except for TBI and earning $100,000 or more per year being associated with increased odds of delaying care due to transportation.

Patients with TBI frequently require complex specialist care, making it particularly concerning that many may fall behind in health literacy and experience financial barriers to care (De Koning et al., 2017; Spaw et al., 2018). Accordingly, targeted strategies to improve and account for lower health literacy among patients with TBI may reduce hospital and emergency room visits (Baker et al., 1998; Cho et al., 2008). Further, particular attention on the part of the clinician to identify gaps in care, whether it be due to low health literacy or financial barriers, and targeted programs to mitigate these obstacles may be particularly helpful among patients with TBI.

Regarding racial disparities among those with TBI, Hispanic and non-Hispanic Black respondents were significantly more likely to report difficulty navigating health material compared to non-Hispanic White respondents. Further, respondents earning less than $35,000 per year were more likely to report issues pertaining to health literacy. These findings were also demonstrated among those without TBI, indicating that these disparities are reflective of a broader issue and not specific to TBI. Additionally, TBI did not consistently modify these effects of race/ethnicity or income, suggesting that TBI may represent an additional, independent burden. These findings are particularly concerning given that Black and Hispanic patients with TBI have been identified to be less likely to be placed into rehabilitative care (Meagher et al., 2015; Shafi et al., 2007). Further, Black and Hispanic patients are more likely to struggle attaining a TBI diagnosis, more likely to have longer wait times in the emergency department, and overall may be more likely to have worse functional outcomes (Gary et al., 2009; Saadi et al., 2022). Being uninsured or on public insurance has been associated with worse functional status even after completing rehabilitation, and such patients are less likely to get placed in rehabilitation (Heffernan et al., 2011; Lequerica et al., 2023). Accordingly, though any single factor is unlikely to explain or resolve these disparities, interrogating these discrepancies from multiple angles and being aware of them are necessary steps for eliminating them.

Financial barriers to medical care represent a substantial challenge for individuals with TBI. Our findings show that these barriers are more commonly reported among non-Hispanic Black and Hispanic respondents with TBI. These findings are consistent with prior work showing that Black and Hispanic patients are less likely to be discharged to rehabilitation following TBI (Bowman et al., 2007) and that racial and ethnic differences in healthcare utilization are partly explained by unequal financial burdens and insurance coverage (Gourevitch et al., 2025; Roberts et al., 2023). Together, this evidence underscores the role of financial barriers in shaping post-TBI care access and utilization.

Overall, although TBI did not consistently modify the effects of race/ethnicity and income in relation to health literacy and access, those with TBI may be uniquely susceptible to issues related to low health literacy and numerous financial barriers to care, as suggested by the self-reported survey responses, with non-Hispanic White and higher income respondents, both with and without history of TBI, being less likely to report these issues. With TBI being a potentially lifelong illness, access to care and literacy surrounding healthcare can make a difference in the long-term health outcomes of patients. With millions of Americans affected by TBI and its extensive financial burden, delay in care due to the costs could be an indirect factor in mortality for patients with TBI (Abdelmalik et al., 2019). Thus, targeting these barriers, particularly in improving organizational health literacy, which refers to an organizational effort to make navigating, understanding, and utilizing care more accessible, may make the healthcare system less burdensome and may be a key part of improving patient outcomes, especially for those with TBI (Brega et al., 2019; Bremer et al., 2021; Farmanova et al., 2018).

### Limitations

There are several important limitations to this investigation. First, many components of All of Us data are self-reported by participants and may not align with the overall demographics of the United States (Mapes et al., 2020), particularly given the lack of survey weighting. Similarly, All of Us is a self-enrolled program, which may limit generalizability of the findings. However, within the enrolled cohort, propensity score matching was applied to minimize measured confounding between participants with and without TBI and allows for potentially useful insights from intragroup comparisons.

Additionally, there is no available data on how long after the initial TBI diagnosis survey results were reported. Therefore, this study only looks at association, not causation. It is possible that patients with low health literacy were at higher initial risk of TBI. Moreover, since, in this analysis, TBI diagnosis is based on affiliated EHR data, there are likely undiagnosed patients with TBI included in the non-TBI cohort. Similarly, conversion of survey responses to SNOMED codes may introduce misclassification error. While such error is expected to be nondifferential between groups, it could attenuate observed associations. Longitudinal analysis comparing health literacy and access to care before and after TBI, with validated diagnosis coding, is necessary to further interrogate how health literacy and access act as predisposing factors versus the consequences of TBI.

Finally, this analysis is limited by the absence of fully validated instruments for measuring health literacy in the All of Us surveys. While there is notable overlap between All of Us survey items and established domains of social determinants of health (Tesfaye et al., 2024), the lack of validated measures constrains external validity. Nevertheless, within-cohort comparisons remain informative, as they highlight how respondents with and without TBI differ in self-reported ability to navigate health materials and in experiencing financial barriers to care, which are areas that have received minimal prior investigation among patients with TBI.

## Conclusion

In summary, our investigation of participants in the NIH's All of Us database revealed that EHR history of TBI is associated with reporting survey responses related to lower health literacy and access to care. Hispanic and non-Hispanic Black respondents, both with and without history of TBI, were more likely to report issues related to health literacy and financial barriers to care, though history of TBI did not modify these effects. Further studies exploring the relationship between TBI, health literacy, and access to healthcare are needed to understand the epidemiology and socioeconomic impact of TBI. Nonetheless, this analysis highlights the need for targeted efforts to improve health literacy and healthcare access for patients with TBI.

## Supplemental Material

sj-docx-1-nre-10.1177_10538135251393519 - Supplemental material for Health Literacy and Access to Care among Patients with and Without Traumatic Brain Injury: An All of Us Analysis Supplemental material, sj-docx-1-nre-10.1177_10538135251393519 for Health Literacy and Access to Care among Patients with and Without Traumatic Brain Injury: An All of Us Analysis by Richard Cook, Kathleen R Ran, Vikas N Vattipally, Gorbachev Jowah, Naren Gundapaneni, Rajiv Dharnipragada, Jose Suarez, John Williams and Tej Azad in NeuroRehabilitation
