# Supplementary material for: Health Literacy and Access to Care among Patients with and Without Traumatic Brain Injury: An All of Us Analysis
Source: NeuroRehabilitation. 2025 Nov 11;58(1):62–72. doi: 10.1177/10538135251393519 (PMC12886559; doi:10.1177/10538135251393519)
Supplement: sj-docx-1-nre-10.1177_10538135251393519 - Supplemental material for Health Literacy and Access to Care among Patients with and Without Traumatic Brain Injury: An All of Us Analysis [file sj-docx-1-nre-10.1177_10538135251393519.docx]

**Supplemental Tables**

**Supplemental Table 1.** Summary of informative nonresponses after matching based on respondents with and without TBI.

| **Variable** | **Respondents with TBI** | **Respondents without TBI** |
| --- | --- | --- |
| Can’t afford dental care (%) | 6.8 | 8.3 |
| Can’t afford emergency care (%) | 5.2 | 6.5 |
| Can’t afford follow-up care (%) | 12.4 | 13.8 |
| Can’t afford a healthcare provider (%) | 11.3 | 13.0 |
| Can’t afford specialist care (%) | 12.1 | 13.3 |
| Confidence (%) | 0.6 | 0.8 |
| Delayed care due to childcare (%) | 12.1 | 14.2 |
| Delayed care due to copay (%) | 12.1 | 13.6 |
| Delayed care due to deductible (%) | 12.7 | 14.8 |
| Delayed care due to paying out of pocket (%) | 12.7 | 14.0 |
| Delayed care due to time off (%) | 7.6 | 9.2 |
| Delayed care due to transportation (%) | 1.7 | 1.7 |
| Delayed medication (%) | 2.9 | 3.4 |
| Difficulty understanding (%) | 1.0 | 1.2 |
| Education (%) | 1.0 | 0.9 |
| Emotion (%) | 0.7 | 0.8 |
| Employment (%) | 7.2 | 7.2 |
| Homeowner status (%) | 2.3 | 2.4 |
| Income (%) | 10.8 | 10.8 |
| Marital status (%) | 1.5 | 1.5 |
| Require assistance (%) | 0.6 | 1.1 |
| Race/ethnicity (%) | 0.0 | 0.0 |
| Sex (%) | 0.0 | 0.0 |
| Skipped medication (%) | 1.0 | 1.5 |
| Took less medications (%) | 1.6 | 2.2 |
| Worried about paying (%) | 2.6 | 3.1 |

**Supplemental Table 2.** Association of TBI with difficulty navigating health material and access to care as determined by multivariable ordinal and binary logistic regression among matched respondents with and without TBI, with results varying based on reclassification of informative nonresponses as either least or most severe responses.

| **Variable** | **Informative nonresponse reclassification** | **OR** | **95% Confidence Interval** | | **P-Value** |
| --- | --- | --- | --- | --- | --- |
| **Difficulty understanding** | Least severe | 1.30 | 1.17 | 1.45 | *< 0.001* |
|  | Most severe | 1.30 | 1.17 | 1.44 | *< 0.001* |
| **Require assistance** | Least severe | 1.23 | 1.11 | 1.35 | *< 0.001* |
|  | Most severe | 1.26 | 1.14 | 1.38 | *< 0.001* |
| **Confidence** | Least severe | 0.89 | 0.80 | 0.98 | 0.019 |
|  | Most severe | 0.91 | 0.82 | 1.01 | 0.068 |
| **Can’t afford…** | | | | | |
| *emergency care* | Least severe | 1.41 | 1.23 | 1.62 | *< 0.001* |
|  | Most severe | 1.55 | 1.26 | 1.91 | *< 0.001* |
| *follow-up care* | Least severe | 1.17 | 1.05 | 1.30 | *0.005* |
|  | Most severe | 1.16 | 0.98 | 1.37 | 0.078 |
| *dental care* | Least severe | 1.12 | 1.01 | 1.24 | *0.031* |
|  | Most severe | 1.03 | 0.91 | 1.16 | 0.634 |
| *a healthcare provider* | Least severe | 1.12 | 1.00 | 1.26 | 0.055 |
|  | Most severe | 0.99 | 0.82 | 1.20 | 0.915 |
| *a specialist* | Least severe | 1.14 | 1.03 | 1.27 | 0.014 |
|  | Most severe | 1.12 | 0.96 | 1.30 | 0.153 |
| **Delayed care due to…** | | | | | |
| *copay* | Least severe | 1.14 | 1.03 | 1.27 | 0.016 |
|  | Most severe | 1.06 | 0.91 | 1.24 | 0.459 |
| *childcare* | Least severe | 1.25 | 1.11 | 1.41 | *< 0.001* |
|  | Most severe | 1.28 | 0.99 | 1.65 | 0.064 |
| *deductible* | Least severe | 1.13 | 1.02 | 1.25 | 0.025 |
|  | Most severe | 1.01 | 0.87 | 1.18 | 0.899 |
| *paying out of pocket* | Least severe | 1.04 | 0.95 | 1.15 | 0.418 |
|  | Most severe | 0.94 | 0.83 | 1.07 | 0.336 |
| *time off work* | Least severe | 1.17 | 1.05 | 1.32 | 0.005 |
|  | Most severe | 1.10 | 0.94 | 1.28 | 0.245 |
| *transportation* | Least severe | 1.37 | 1.19 | 1.58 | *< 0.001* |
|  | Most severe | 1.48 | 1.27 | 1.73 | *< 0.001* |
| **Took less medications** | Least severe | 1.21 | 1.05 | 1.38 | 0.007 |
|  | Most severe | 1.16 | 1.00 | 1.35 | 0.059 |
| **Delayed medications** | Least severe | 1.23 | 1.09 | 1.38 | *< 0.001* |
|  | Most severe | 1.22 | 1.07 | 1.39 | *0.003* |
| **Skipped medications** | Least severe | 1.27 | 1.10 | 1.47 | *< 0.001* |
|  | Most severe | 1.26 | 1.08 | 1.48 | 0.004 |
| **Worried about paying** | Least severe | 1.04 | 0.91 | 1.20 | 0.557 |
|  | Most severe | 1.09 | 0.96 | 1.23 | 0.210 |

**Supplemental Table 3.** Difficulty navigating health material and access to various aspects of healthcare by race and ethnicity among participants with TBI, with results varying based on reclassification of informative nonresponses as either least or most severe responses (intermediate responses are unaffected, and thus not shown).

| **Variable** | **Informative nonresponse reclassification** | **Black, non-Hispanic (174)** | **Hispanic (175)** | **White, non-Hispanic (1,734)** | **P-Value** |
| --- | --- | --- | --- | --- | --- |
| **Difficulty Understanding (%)** | | | | | |
| *Never* | Least severe | 61.5 | 67.4 | 75.7 | *< 0.001* |
|  | Most severe | 61.5 | 65.7 | 74.7 | *< 0.001* |
| *Always* | Least severe | 3.4 | 5.1 | 0.5 | *< 0.001* |
|  | Most severe | 3.4 | 6.9 | 1.4 | *< 0.001* |
| **Assistance with Medical Forms (%)** | | | | | |
| *Never* | Least severe | 62.6 | 56.6 | 71.3 | *< 0.001* |
|  | Most severe | 60.9 | 56.0 | 70.4 | *< 0.001* |
| *Always* | Least severe | 2.9 | 8.6 | 1.0 | *< 0.001* |
|  | Most severe | 4.6 | 9.1 | 1.9 | *< 0.001* |
| **Confidence with Medical Forms (%)** | | | | | |
| *Not at all* | Least severe | 1.7 | 1.7 | 1.2 | 0.508 |
|  | Most severe | 1.1 | 1.7 | 0.5 | 0.085 |
| *Extremely* | Least severe | 66.7 | 59.4 | 74.7 | *< 0.001* |
|  | Most severe | 67.2 | 59.4 | 75.4 | *< 0.001* |
| **Can’t afford… (%)** | | | | | |
| *emergency care* | Least severe | 9.2 | 10.3 | 5.1 | 0.004 |
|  | Most severe | 19.0 | 17.1 | 11.3 | *0.002* |
| *follow-up* | Least severe | 12.6 | 16.6 | 7.6 | *< 0.001* |
|  | Most severe | 30.5 | 28.0 | 21.0 | *0.003* |
| *a healthcare provider* | Least severe | 13.2 | 10.9 | 5.3 | *< 0.001* |
|  | Most severe | 28.7 | 22.9 | 17.5 | *0.001* |
| *dental care* | Least severe | 23.6 | 25.1 | 17.9 | 0.018 |
|  | Most severe | 36.2 | 32.0 | 25.8 | 0.004 |
| *specialist care* | Least severe | 14.4 | 18.9 | 9.6 | *< 0.001* |
|  | Most severe | 32.8 | 29.7 | 22.3 | *0.001* |
| **Delayed care due to… (%)** | | | | | |
| *copay* | Least severe | 12.1 | 15.4 | 9.3 | 0.023 |
|  | Most severe | 27.6 | 27.4 | 22.8 | 0.173 |
| *childcare* | Least severe | 5.7 | 8.6 | 2.8 | *< 0.001* |
|  | Most severe | 21.8 | 19.4 | 17.0 | 0.227 |
| *deductible* | Least severe | 10.9 | 15.4 | 10.0 | 0.085 |
|  | Most severe | 28.2 | 29.1 | 24.8 | 0.313 |
| *paying out of pocket* | Least severe | 14.9 | 18.3 | 17.1 | 0.690 |
|  | Most severe | 32.2 | 31.4 | 31.2 | 0.964 |
| *time off work* | Least severe | 11.5 | 18.3 | 10.3 | 0.006 |
|  | Most severe | 24.7 | 29.1 | 19.1 | *0.002* |
| *transportation* | Least severe | 22.4 | 16.0 | 9.5 | *< 0.001* |
|  | Most severe | 28.2 | 18.9 | 10.7 | *< 0.001* |
| **Worried about paying (%)** | | | | | |
| *Very* | Least severe | 14.4 | 26.3 | 11.6 | *< 0.001* |
|  | Most severe | 21.8 | 29.1 | 13.7 | *< 0.001* |
| **Took less medication** | Least severe | 12.6 | 15.4 | 10.4 | 0.097 |
|  | Most severe | 18.4 | 20.6 | 12.1 | *0.001* |
| **Skipped medication** | Least severe | 12.1 | 14.3 | 9.5 | 0.093 |
|  | Most severe | 15.5 | 16.0 | 10.6 | 0.020 |
| **Delayed medication** | Least severe | 17.8 | 17.7 | 15.1 | 0.442 |
|  | Most severe | 23.6 | 21.1 | 18.3 | 0.177 |

**Supplemental Table 4.** Difficulty navigating health material and access to various aspects of healthcare by race and ethnicity among participants without TBI.

| **Variable** | **Black, non-Hispanic (1,005)** | **Hispanic (996)** | **White, non-Hispanic (9,561)** | **P-Value** |
| --- | --- | --- | --- | --- |
| **Difficulty Understanding (%)** | | | | |
| *Never* | 65.1 | 72.4 | 78.4 | *< 0.001* |
| *Occasionally* | 12.4 | 15.2 | 14.6 | 0.149 |
| *Sometimes* | 14.4 | 9.0 | 4.6 | *< 0.001* |
| *Often* | 3.0 | 1.5 | 1.1 | *< 0.001* |
| *Always* | 3.3 | 1.3 | 0.4 | *< 0.001* |
| *Skip* | 1.8 | 0.6 | 1.0 | 0.018 |
| **Assistance with Medical Forms (%)** | | | | |
| *Never* | 62.5 | 66.4 | 73.1 | *< 0.001* |
| *Occasionally* | 14.4 | 17.9 | 17.2 | 0.063 |
| *Sometimes* | 14.4 | 10.3 | 6.0 | *< 0.001* |
| *Often* | 3.1 | 3.3 | 2.0 | 0.004 |
| *Always* | 4.2 | 1.9 | 1.1 | *< 0.001* |
| *Skip* | 1.4 | 0.2 | 0.6 | *0.002* |
| **Confidence with Medical Forms (%)** | | | | |
| *Not at all* | 1.9 | 0.8 | 0.3 | *< 0.001* |
| *A little bit* | 3.0 | 0.8 | 0.5 | *< 0.001* |
| *Somewhat* | 10.5 | 7.8 | 4.3 | *< 0.001* |
| *Quite a bit* | 17.3 | 24.9 | 19.2 | *< 0.001* |
| *Extremely* | 66.1 | 65.6 | 75.1 | *< 0.001* |
| *Skip* | 1.2 | 0.1 | 0.6 | 0.005 |
| **Can’t afford… (%)** | | | | |
| *emergency care* | 6.0 | 7.0 | 3.2 | *< 0.001* |
| *follow-up* | 11.3 | 11.3 | 6.4 | *< 0.001* |
| *a healthcare provider* | 9.9 | 10.6 | 5.4 | *< 0.001* |
| *dental care* | 22.1 | 28.5 | 16.6 | *< 0.001* |
| *specialist care* | 12.6 | 14.4 | 8.5 | *< 0.001* |
| **Delayed care due to… (%)** | | | | |
| *copay* | 10.7 | 15.9 | 8.3 | *< 0.001* |
| *childcare* | 3.9 | 6.0 | 2.3 | *< 0.001* |
| *deductible* | 8.7 | 14.7 | 9.8 | *< 0.001* |
| *paying out of pocket* | 15.7 | 24.0 | 16.7 | *< 0.001* |
| *time off work* | 9.8 | 19.0 | 9.1 | *< 0.001* |
| *transportation* | 13.9 | 15.7 | 6.7 | *< 0.001* |
| **Worried about paying (%)** | | | | |
| *Somewhat* | 32.9 | 39.1 | 37.2 | 0.011 |
| *Very* | 15.4 | 22.1 | 11.0 | *< 0.001* |
| **Took less medication** | 9.3 | 12.4 | 8.8 | *0.001* |
| **Skipped medication** | 9.0 | 11.0 | 7.4 | *< 0.001* |
| **Delayed medication** | 14.6 | 17.5 | 11.9 | *< 0.001* |

**Supplemental Table 5.** Difficulty navigating health material by income among participants with TBI, with results varying based on reclassification of informative nonresponses as either least or most severe responses (intermediate responses are unaffected, and thus not shown).

| **Variable** | **Informative nonresponse reclassification** | | **< $35,000/yr (657)** | **$35,000-74,999/yr (583)** | **$75,000-99,999/yr (261)** | **≥ $100,000/yr (582**) | **P-Value** |
| --- | --- | --- | --- | --- | --- | --- | --- |
| **Difficulty Understanding (%)** | | | | | | | |
| *Never* | Least severe | 59.1 | | 75.6 | 78.9 | 86.3 | *< 0.001* |
|  | Most severe | 58.0 | | 74.6 | 77.8 | 85.6 | *< 0.001* |
| *Always* | Least severe | 2.6 | | 0.7 | 0.4 | 0.2 | *< 0.001* |
|  | Most severe | 3.7 | | 1.7 | 1.5 | 0.9 | 0.004 |
| **Assistance with Medical Forms (%)** | | | | | | | |
| *Never* | Least severe | 54.9 | | 72.2 | 77.4 | 79.0 | *< 0.001* |
|  | Most severe | 53.9 | | 71.2 | 76.6 | 78.4 | *< 0.001* |
| *Always* | Least severe | 4.0 | | 1.0 | 1.1 | 0.5 | *< 0.001* |
|  | Most severe | 5.0 | | 2.1 | 1.9 | 1.2 | *< 0.001* |
| **Confidence with Medical Forms (%)** | | | | | | | |
| *Not at all* | Least severe | 2.0 | | 1.5 | 0.8 | 0.3 | 0.040 |
|  | Most severe | 1.2 | | 0.9 | 0.0 | 0.2 | 0.063 |
| *Extremely* | Least severe | 60.0 | | 73.1 | 75.9 | 85.6 | *< 0.001* |
|  | Most severe | 60.7 | | 73.8 | 76.6 | 85.7 | *< 0.001* |

**Supplemental Table 6.** Difficulty navigating health material by income among participants without TBI.

| **Variable** | **< $35,000/yr (3,211)** | **$35,000-74,999/yr (2,925)** | **$75,000-99,999/yr (1,233)** | **≥ $100,000/yr (2,945**) | **P-value** |
| --- | --- | --- | --- | --- | --- |
| **Difficulty Understanding (%)** | | | | | |
| *Never* | 66.0 | 77.5 | 83.5 | 87.4 | *< 0.001* |
| *Occasionally* | 18.0 | 16.2 | 12.9 | 9.6 | *< 0.001* |
| *Sometimes* | 11.1 | 4.3 | 2.4 | 1.8 | *< 0.001* |
| *Often* | 2.4 | 0.8 | 0.4 | 0.3 | *< 0.001* |
| *Always* | 1.4 | 0.3 | 0.2 | 0.0 | *< 0.001* |
| *Skip* | 1.2 | 0.8 | 0.6 | 0.9 | 0.265 |
| **Assistance with Medical Forms (%)** | | | | | |
| *Never* | 63.0 | 73.5 | 76.2 | 80.4 | *< 0.001* |
| *Occasionally* | 18.9 | 17.8 | 17.1 | 14.3 | *< 0.001* |
| *Sometimes* | 11.4 | 5.5 | 4.3 | 3.2 | *< 0.001* |
| *Often* | 3.4 | 1.8 | 1.5 | 1.2 | *< 0.001* |
| *Always* | 2.6 | 0.9 | 0.3 | 0.5 | *< 0.001* |
| *Skip* | 0.8 | 0.4 | 0.6 | 0.4 | 0.054 |
| **Confidence with Medical Forms (%)** | | | | | |
| *Not at all* | 1.1 | 0.2 | 0.2 | 0.0 | *< 0.001* |
| *A little bit* | 1.7 | 0.4 | 0.1 | 0.1 | *< 0.001* |
| *Somewhat* | 9.9 | 3.9 | 1.9 | 1.3 | *< 0.001* |
| *Quite a bit* | 25.4 | 21.1 | 17.3 | 12.9 | *< 0.001* |
| *Extremely* | 61.3 | 74.1 | 80.1 | 85.2 | *< 0.001* |
| *Skip* | 0.6 | 0.4 | 0.4 | 0.5 | 0.642 |

**Supplemental Table 7.** Association of interaction between TBI and race/ethnicity with difficulty navigating health material and access to care as determined by multivariable ordinal logistic regression among matched participants with and without TBI.

| **Variable** | **Interaction term (TBI * …)** | **OR** | **95% Confidence Interval** | | **P-Value** |
| --- | --- | --- | --- | --- | --- |
| **Difficulty understanding** | non-Hispanic Black | 0.88 | 0.56 | 1.39 | 0.596 |
|  | non-Hispanic White | 0.83 | 0.58 | 1.17 | 0.285 |
| **Require assistance** | non-Hispanic Black | 0.74 | 0.48 | 1.15 | 0.184 |
|  | non-Hispanic White | 0.74 | 0.54 | 1.02 | 0.067 |
| **Confidence** | non-Hispanic Black | 0.99 | 0.63 | 1.56 | 0.974 |
|  | non-Hispanic White | 1.20 | 0.86 | 1.67 | 0.290 |
| **Can’t afford…** | | | | | |
| *emergency care* | non-Hispanic Black | 1.05 | 0.48 | 2.29 | 0.900 |
|  | non-Hispanic White | 1.07 | 0.6 | 1.94 | 0.813 |
| *follow-up care* | non-Hispanic Black | 0.65 | 0.34 | 1.26 | 0.201 |
|  | non-Hispanic White | 0.74 | 0.46 | 1.19 | 0.218 |
| *dental care* | non-Hispanic Black | 1.26 | 0.75 | 2.13 | 0.379 |
|  | non-Hispanic White | 1.40 | 0.95 | 2.05 | 0.085 |
| *a healthcare provider* | non-Hispanic Black | 1.20 | 0.6 | 2.42 | 0.602 |
|  | non-Hispanic White | 0.95 | 0.55 | 1.63 | 0.854 |
| *a specialist* | non-Hispanic Black | 0.76 | 0.41 | 1.40 | 0.376 |
|  | non-Hispanic White | 0.76 | 0.49 | 1.18 | 0.228 |
| **Delayed care due to…** | | | | | |
| *copay* | non-Hispanic Black | 1.18 | 0.61 | 2.28 | 0.615 |
|  | non-Hispanic White | 1.10 | 0.69 | 1.77 | 0.680 |
| *childcare* | non-Hispanic Black | 1.57 | 0.87 | 2.84 | 0.704 |
|  | non-Hispanic White | 0.83 | 0.32 | 2.14 | 0.441 |
| *deductible* | non-Hispanic Black | 1.23 | 0.63 | 2.42 | 0.548 |
|  | non-Hispanic White | 0.90 | 0.56 | 1.44 | 0.648 |
| *paying out of pocket* | non-Hispanic Black | 1.20 | 0.66 | 2.19 | 0.555 |
|  | non-Hispanic White | 1.42 | 0.93 | 2.16 | 0.104 |
| *time off work* | non-Hispanic Black | 1.27 | 0.64 | 2.51 | 0.500 |
|  | non-Hispanic White | 1.12 | 0.71 | 1.77 | 0.621 |
| *transportation* | non-Hispanic Black | 1.77 | 0.99 | 3.17 | 0.053 |
|  | non-Hispanic White | 1.46 | 0.92 | 2.30 | 0.108 |
| **Took less medications** | non-Hispanic Black | 1.33 | 0.68 | 2.59 | 0.409 |
|  | non-Hispanic White | 1.15 | 0.70 | 1.88 | 0.585 |
| **Delayed medications** | non-Hispanic Black | 1.34 | 0.74 | 2.43 | 0.336 |
|  | non-Hispanic White | 1.43 | 0.91 | 2.23 | 0.117 |
| **Skipped medications** | non-Hispanic Black | 1.16 | 0.58 | 2.32 | 0.672 |
|  | non-Hispanic White | 1.20 | 0.72 | 1.99 | 0.480 |
| **Worried about paying** | non-Hispanic Black | 0.68 | 0.38 | 1.21 | 0.193 |
|  | non-Hispanic White | 0.74 | 0.50 | 1.10 | 0.133 |

**Supplemental Table 8.** Association of interaction between TBI and income with difficulty navigating health material and access to care as determined by multivariable ordinal logistic regression among matched participants with and without TBI.

| **Variable** | **Interaction term (TBI * …)** | **OR** | **95% Confidence Interval** | | **P-Value** |
| --- | --- | --- | --- | --- | --- |
| **Difficulty understanding** | $35,000-$74,999/yr | 0.81 | 0.62 | 1.07 | 0.141 |
|  | $75,000-$99,999/yr | 0.96 | 0.66 | 1.40 | 0.836 |
|  | > $100,000/yr | 0.81 | 0.59 | 1.11 | 0.192 |
| **Require assistance** | $35,000-$74,999/yr | 0.77 | 0.59 | 1.00 | 0.051 |
|  | $75,000-$99,999/yr | 0.64 | 0.45 | 0.93 | 0.017 |
|  | > $100,000/yr | 0.75 | 0.57 | 0.99 | 0.039 |
| **Confidence** | $35,000-$74,999/yr | 1.00 | 0.76 | 1.30 | 0.974 |
|  | $75,000-$99,999/yr | 0.86 | 0.59 | 1.24 | 0.414 |
|  | > $100,000/yr | 1.13 | 0.83 | 1.54 | 0.453 |
| **Can’t afford…** | | | | | |
| *emergency care* | $35,000-$74,999/yr | 0.74 | 0.44 | 1.23 | 0.245 |
|  | $75,000-$99,999/yr | 0.66 | 0.28 | 1.53 | 0.329 |
|  | > $100,000/yr | 1.52 | 0.76 | 3.05 | 0.234 |
| *follow-up care* | $35,000-$74,999/yr | 0.92 | 0.62 | 1.38 | 0.692 |
|  | $75,000-$99,999/yr | 0.90 | 0.47 | 1.73 | 0.751 |
|  | > $100,000/yr | 0.78 | 0.44 | 1.39 | 0.400 |
| *dental care* | $35,000-$74,999/yr | 1.15 | 0.86 | 1.54 | 0.348 |
|  | $75,000-$99,999/yr | 1.21 | 0.77 | 1.91 | 0.415 |
|  | > $100,000/yr | 1.30 | 0.88 | 1.93 | 0.192 |
| *a healthcare provider* | $35,000-$74,999/yr | 0.77 | 0.48 | 1.25 | 0.292 |
|  | $75,000-$99,999/yr | 0.77 | 0.37 | 1.62 | 0.490 |
|  | > $100,000/yr | 1.34 | 0.73 | 2.46 | 0.352 |
| *a specialist* | $35,000-$74,999/yr | 0.85 | 0.59 | 1.22 | 0.376 |
|  | $75,000-$99,999/yr | 0.63 | 0.34 | 1.17 | 0.145 |
|  | > $100,000/yr | 0.81 | 0.49 | 1.33 | 0.397 |
| **Delayed care due to…** | | | | | |
| *copay* | $35,000-$74,999/yr | 1.05 | 0.73 | 1.53 | 0.780 |
|  | $75,000-$99,999/yr | 0.93 | 0.51 | 1.69 | 0.802 |
|  | > $100,000/yr | 1.33 | 0.77 | 2.29 | 0.307 |
| *childcare* | $35,000-$74,999/yr | 0.47 | 0.23 | 0.93 | 0.030 |
|  | $75,000-$99,999/yr | 0.37 | 0.13 | 1.10 | 0.073 |
|  | > $100,000/yr | 0.31 | 0.14 | 0.68 | 0.003 |
| *deductible* | $35,000-$74,999/yr | 1.08 | 0.74 | 1.57 | 0.683 |
|  | $75,000-$99,999/yr | 0.74 | 0.42 | 1.30 | 0.288 |
|  | > $100,000/yr | 1.09 | 0.69 | 1.72 | 0.712 |
| *paying out of pocket* | $35,000-$74,999/yr | 1.03 | 0.75 | 1.41 | 0.858 |
|  | $75,000-$99,999/yr | 0.82 | 0.52 | 1.28 | 0.378 |
|  | > $100,000/yr | 1.37 | 0.97 | 1.93 | 0.075 |
| *time off work* | $35,000-$74,999/yr | 1.13 | 0.76 | 1.68 | 0.560 |
|  | $75,000-$99,999/yr | 0.64 | 0.37 | 1.12 | 0.121 |
|  | > $100,000/yr | 0.60 | 0.37 | 0.95 | 0.031 |
| *transportation* | $35,000-$74,999/yr | 0.99 | 0.65 | 1.52 | 0.976 |
|  | $75,000-$99,999/yr | 0.85 | 0.42 | 1.74 | 0.659 |
|  | > $100,000/yr | 2.12 | 1.29 | 3.50 | *0.003* |
| **Took less medications** | $35,000-$74,999/yr | 0.93 | 0.65 | 1.35 | 0.714 |
|  | $75,000-$99,999/yr | 0.95 | 0.55 | 1.61 | 0.836 |
|  | > $100,000/yr | 0.81 | 0.48 | 1.38 | 0.438 |
| **Delayed medications** | $35,000-$74,999/yr | 0.81 | 0.59 | 1.12 | 0.205 |
|  | $75,000-$99,999/yr | 0.95 | 0.60 | 1.51 | 0.840 |
|  | > $100,000/yr | 1.12 | 0.74 | 1.71 | 0.587 |
| **Skipped medications** | $35,000-$74,999/yr | 1.07 | 0.74 | 1.55 | 0.729 |
|  | $75,000-$99,999/yr | 0.81 | 0.44 | 1.50 | 0.502 |
|  | > $100,000/yr | 0.71 | 0.38 | 1.32 | 0.278 |
| **Worried about paying** | $35,000-$74,999/yr | 1.04 | 0.74 | 1.46 | 0.820 |
|  | $75,000-$99,999/yr | 0.91 | 0.53 | 1.57 | 0.745 |
|  | > $100,000/yr | 1.03 | 0.62 | 1.71 | 0.900 |
